# Intrasphenoidal cephalocoeles: Knowing the less known

**DOI:** 10.4102/sajr.v28i1.2829

**Published:** 2024-05-10

**Authors:** Prachi Mann, Sanjog S. Gajbhiye, Deoyani Sarjare, Aarti Anand, Shrikant Kalbagwar, Pramod Giri

**Affiliations:** 1 Department of Radiodiagnosis, Government Medical College, Nagpur, India; 2 Department of Neurosurgery, Government Medical College and Superspeciality Hospital, Nagpur, India

**Keywords:** CSF rhinorrhoea, intrasphenoidal encephalocoeles, spontaneous lateral sphenoid cephalocoeles, SLSC, Sternberg’s canal, lateral craniopharyngeal canal, CT cisternography

## Abstract

Intrasphenoidal encephalocoeles are acquired or congenital herniations of meninges and brain parenchyma through a structural sphenoid bone defect. Acquired causes are most common, either iatrogenic, post-traumatic, or spontaneous. However, defects in the lateral wall of the sphenoid sinus are uncommon and cephalocoeles through them relatively underexplored in current literature, warranting dedicated attention to unravel their complexities. Congenital causes such as persistence of Sternberg’s canal, which can lead to lateral cephalocoeles, is a rare entity, seen in two of the presented cases, based on the location of the defect with respect to the line connecting the foramen rotundum and the vidian canal (VR line). Three cases of intrasphenoidal cephalocoeles are presented; two patients presented with watery nasal discharge without prior trauma or surgery and the third case was incidentally detected in an elderly patient with intraparenchymal haemorrhage. Imaging with CT cisternography and brain MR were performed to ascertain the exact location of the leak and confirm the presence of herniated brain tissue via the defects. Patients were evaluated by otolaryngology for transnasal endoscopic repair, which was deemed unfeasible, and referred to neurosurgery for transcranial duroplasty.

**Contribution:**

These cases provide crucial insights into the aetiology of lateral intrasphenoidal cephalocoeles, offering a practical system to classify cerebrospinal fluid (CSF) leaks based on the bony defect location. The three illustrative cases and emphasis on advanced imaging modalities refine the knowledge of their aetiology, clinical presentation and management, which hold direct clinical relevance for accurate diagnosis and tailored management of these rare anomalies.

## Introduction

Intrasphenoidal encephalocoeles, also termed basal encephalocoeles, represent around 10% of all encephalocoeles.^1^ These are further divided into the medial or perisellar and lateral types.^2^ When these defects are off midline in the sphenoid, they are known as spontaneous lateral sphenoid encephalocoeles (SLSC) and are classified further as type 1 and 2, based on pneumatisation status and presentation.^3^

- Type 1 SLSC: There is a defect in the pneumatised lateral recess of the sphenoid sinus (SS). These patients typically present with cerebrospinal fluid (CSF) rhinorrhoea, headache, or complications such as meningitis and abscess.
- Type 2 SLSC: This occurs due to osseous dehiscence in the greater wing of sphenoid. There is no herniation within the SS in this subtype. These patients most commonly present with seizures, headaches or incidentally.

**FIGURE 1:**
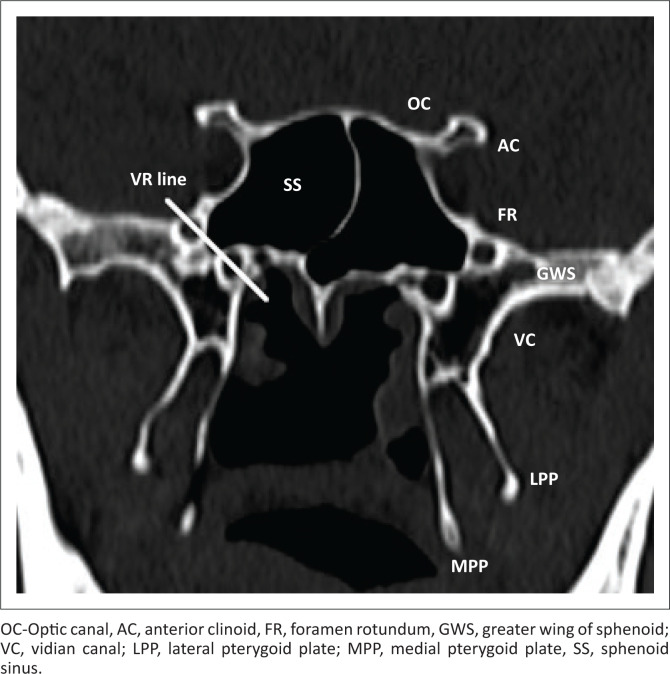
Coronal view of the normal sphenoid sinus anatomy.

**FIGURE 2:**
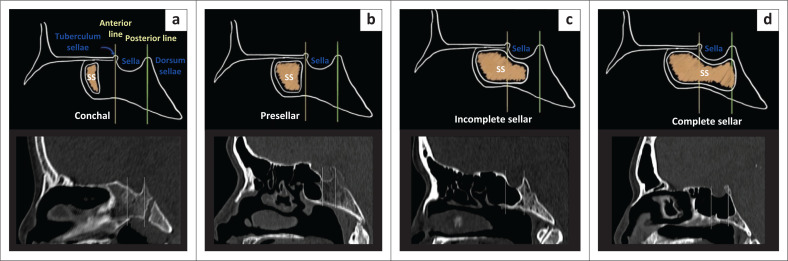
Illustration depicting types of sphenoid pneumatisation. Landmarks-anterior line perpendicular through tuberculum sella and posterior through dorsum sellae. (a) conchal type; (b) presellar; (c) incomplete sellar (reaching the posterior line); (d) complete sellar (crossing the posterior line).

Embryologically, the basis of intrasphenoidal encephalocoeles lies in understanding the development of the SS and its pneumatisation, which is highly variable. The sphenoid bone is formed by seven pairs of ossification centres, which appear by the fourth month of foetal life and undergo fusion and invagination by the nasal mucosa and further pneumatisation to develop completely by adolescence. The fusion planes between these centres of ossification offer resistance to pneumatisation and form septae and crests within it. The normal anatomy of the SS and different types of pneumatisation are depicted in Figure 1 and Figure 2.

**FIGURE 3:**
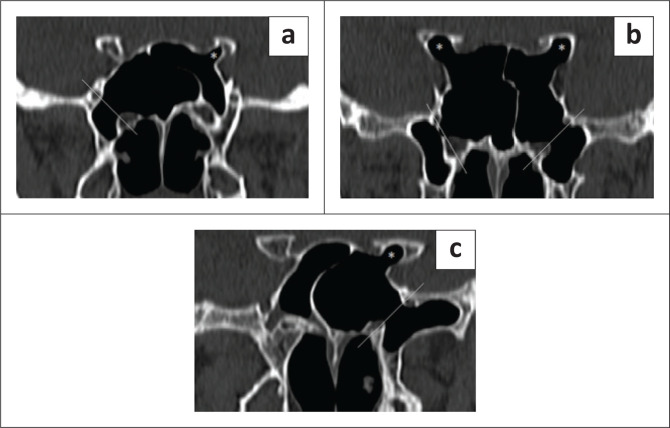
Different combinations of lateral pneumatisation of the sphenoid. (a) Pneumatisation of the right pterygoid and left anterior clinoid process. (*) Marking the anterior clinoid process. (b) Bilateral pterygoid and anterior clinoid processes. (c) Full lateral-left greater wing, pterygoid and anterior clinoid pneumatisation.

Lateral extension of pneumatisation is classified into the greater wing of the sphenoid, pterygoid, and full lateral (greater wing and pterygoid) extension based on the VR line (an imaginary line connecting the foramen rotundum [FR] and vidian canal [VC] in coronal section)^4^ (Figure 3). The lesser wing extension is said to be present when there is an extension into either optic strut, lesser wing, or the anterior clinoid processes.

The extensive laterality of pneumatisation of SS into the greater wing of sphenoid and pterygoid plates results in the formation of a lateral recess, which is a potential weak site, prone to the development of osseous defects and subsequent brain tissue herniation. This, along with increased intracranial CSF pressure, predisposes to herniation of the dura, arachnoid and brain matter through the osseous defects in the walls of the SS.^3^

**FIGURE 4:**
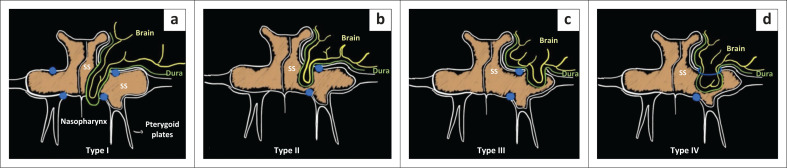
Based on the vidian canal (VR line), there are four leak types: (a) Type I (medial to foramen rotundum [FR] and vidian canal [VC] entering the nasopharynx) via Sternberg’s canal. (b) Type II is medial to the FR. (c) Type III is lateral to the FR. (d) Type IV passes through an enlarged FR into the sphenoid.

The classification of the four types of CSF leaks is dependant upon the site of the leak through the lateral wall of the SS^5,6^ (Figure 4):

- Type I (medial to FR and VC entering the nasopharynx) via Sternberg’s canal.
- Type II medial to FR.
- Type III lateral to FR.
- Type IV through an enlarged FR into the sphenoid.

Congenital aetiologies are rare, but may occur because of persistence of the hypophyseal canal or craniopharyngeal canal that leads to a midline defect in the skull base causing a transsphenoidal encephalocoele, or herniation of the sellar-suprasellar contents, presenting as a nasopharyngeal soft tissue mass. They may also occur as a result of persistence of Sternberg’s canal or the lateral craniopharyngeal canal (medial to the VR line) causing lateral intrasphenoidal encephalocoeles.

This case series describes three illustrative cases of intrasphenoidal cephalocoeles and classifies them according to the location of bony defects and type of CSF leak.

## Case series

### Case 1

**FIGURE 5:**
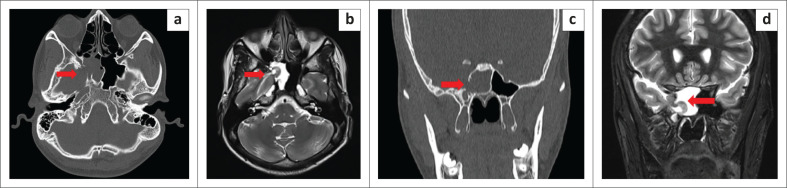
(a and c) Axial and coronal CT sections demonstrate a defect in the right lateral wall of the sphenoid sinus (red arrows). (b and d) Axial and coronal T2-weighted sequences reveal the herniated portion of right medial temporal lobe (red arrows).

**FIGURE 6:**
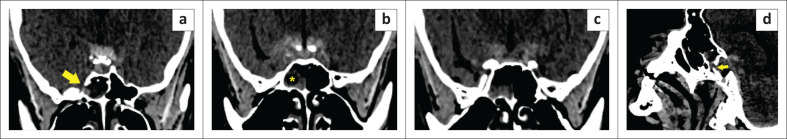
CT cisternography coronal and sagittal reformatted images. (a and d) Demonstrate a defect in the right lateral wall of the sphenoid (yellow arrow). (b and c) Opacification of sphenoid sinus, (*) The herniated temporal lobe.

**FIGURE 7:**
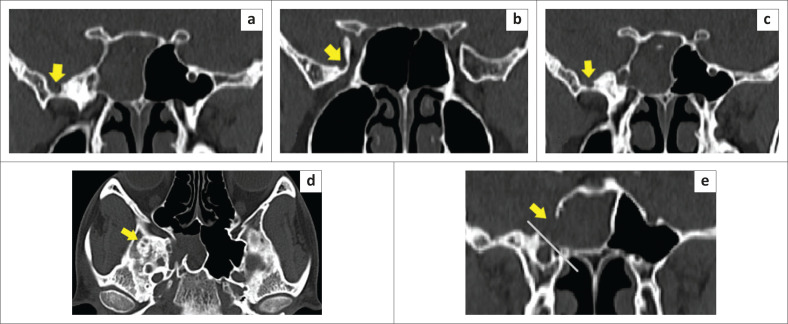
(a, b, c, d) Shows multiple arachnoid pits in the right greater wing of sphenoid. (e) Defect medial to the VR line in the location of Sternberg’s canal suggestive of a Type 1 SLSC with a Type I leak. (yellow arrows indicate the arachnoid pits and osseous sphenoid defects).

**FIGURE 8:**
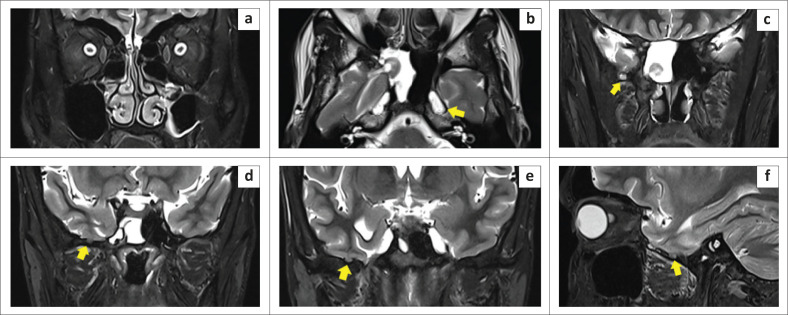
Signs of intracranial hypertension. (a) Prominent subarachnoid spaces around both optic nerves. (b) Enlarged Meckel’s caves bilaterally. (c, d, e, f) Multiple arachnoid pits with tiny meningocoeles (yellow arrows).

A 25-year-old male with a 1-month history of salty watery discharge from the right nostril and headache for 10 days was referred to the otorhinolaryngology department. Physical examination revealed a watery discharge from the right nasal vestibule on forward bending and straining. There was no history of prior head trauma, surgery, or infection. The CSF analysis was normal with no evidence of meningitis. Non-contrast CT of the paranasal sinuses (PNS) revealed a defect in the right lateral wall of the SS with pneumatisation of the lateral recesses (Figure 5a and c). Imaging with CT cisternography revealed opacification of the right half of the SS with hyperdense fluid from the subarachnoid space of the middle cranial fossa surrounding the herniated brain tissue (Figure 6). Brain MRI demonstrated herniation of the right medial temporal lobe through the bony defect into the right lateral recess of the SS (Figure 5b and d). Multiple tiny arachnoid pits were seen in the greater wing of sphenoid on the right side (Figure 7). Signs of increased intracranial pressure, namely prominence of the subarachnoid spaces around both optic nerves, enlarged Meckel’s caves and prominent arachnoid pits were present (Figure 8). The case was classified as Type 1 SLSC with a Type I CSF leak (medial to the VR line).

**FIGURE 9:**
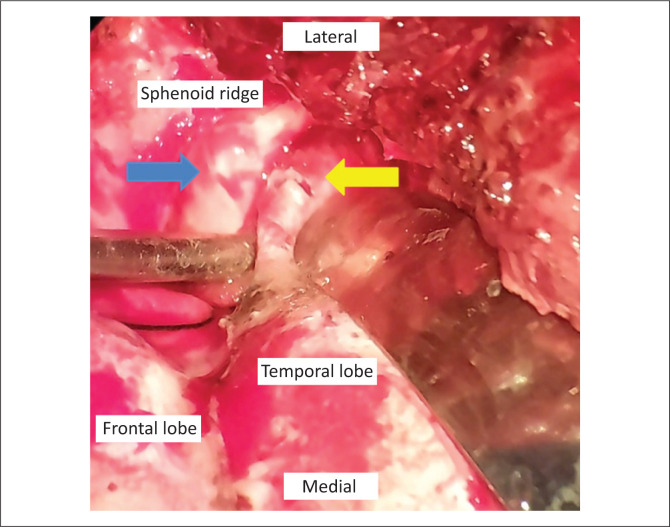
Intraoperative photograph showing the osseous defect (blue arrow) in the sphenoid sinus and temporal dural defect (yellow arrow).

As a result of the defect’s far lateral placement, which made endoscopic access challenging, the patient was referred to neurosurgery for transcranial repair of the defect with duroplasty. A right pterional craniotomy was performed, followed by extradural dissection to identify the bony defect and the temporal lobe encephalocoele (Figure 9). The herniated temporal lobe was gliotic and therefore excised. The dural defect was sutured with an onlay autologous pericranial locally harvested graft and the bony defect was secured with a temporalis myofascial graft. No glue was used. No recurrence of the CSF leakage had occurred at follow-up 7 months post-surgery.

### Case 2

**FIGURE 10:**
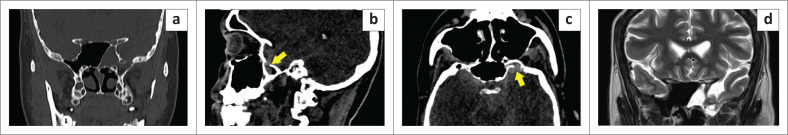
(a) Coronal reformatted CT reveals a bony defect in the roof of the left lateral wall, lateral to the foramen rotundum – Type 1 spontaneous lateral sphenoid cephalocoele (SLSC) with a type III leak. (b and c) CT cisternography sagittal and axial reformatted images show contrast leakage from the subarachnoid space to the left half of the sphenoid sinus. (d) Coronal T2-weighted sequence shows herniated brain tissue within the sphenoid sinus.

A 36-year-old male presented with a 4-day history of spontaneous watery discharge from the left nostril, along with fever and headache. No history of any previous head trauma or surgery was present. On examination, there was no neck rigidity and the CSF analysis was normal. Non-contrast-enhanced CT of the PNS revealed a defect in the roof of left lateral wall of the SS with thinning of the greater wing and full lateral sphenoid pneumatisation (Figure 10A). CT cisternography revealed opacification of the left half of the SS (Figure 10b and c) and MRI confirmed herniation of the medial temporal lobe through the bony defect (Figure 10d) which was classified as a Type 1 SLSC with a type III CSF leak (lateral to VR line). A transcranial extradural onlay patch approach (as described in case 1) was used to repair the defect. The patient had no recurrent CSF rhinorrhoea.

### Case 3

**FIGURE 11:**
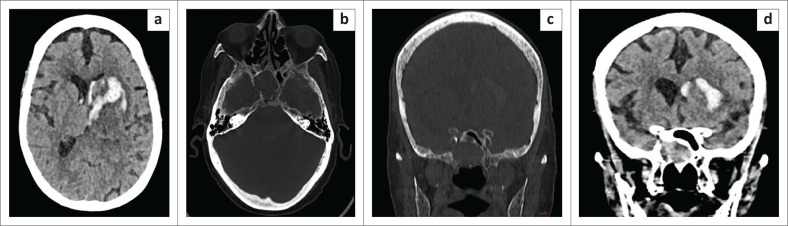
(a) Axial CT reveals intraparenchymal haemorrhage in the left gangliocapsular region with intraventricular extension. (b and c) Axial and Coronal CT demonstrates the defect in the right lateral wall of sphenoid sinus. (d) Herniated brain tissue within the sphenoid.

**FIGURE 12:**
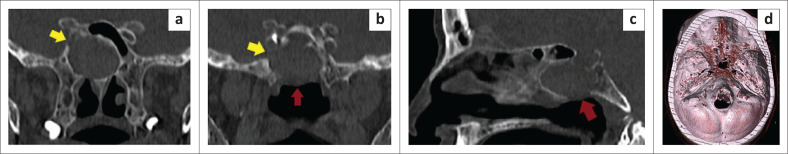
(a) Defect medial to the vidian canal (VR line) without extension of pneumatisation into the pterygoid or greater wing – type I leak. (b and c) Dehiscent floor of the sphenoid sinus. (d) Surface shaded display reveals the defect in the floor of sphenoid sinus communicating with the nasopharynx.

An 84-year-old hypertensive female presented to the emergency department with altered sensorium and right-sided weakness on the same morning. Brain CT revealed intraparenchymal haemorrhage in the left gangliocapsular region with intra ventricular extension. Incidental note of a 7mm bone defect in the right lateral wall and floor of the SS with herniation of brain tissue (Figure 11 and Figure 12) was made. It was classified as a transsphenoidal cephalocoele with a Type I CSF leak (medial to the VR line with extension into the nasopharynx). The patient was managed conservatively.

## Discussion

The two main factors that play an important role in the development of spontaneous intrasphenoidal encephalocoeles are altered CSF dynamics and osseous thinning.^3^ In this series, case 1 and case 3 are typical examples of CSF leaks from basal-temporal encephalocoeles because of persistence of Sternberg’s canal while case 2 is an example of a CSF leak from a basal-temporal encephalocoele as a result of extensive lateral pneumatisation of the SS.

Patients with intrasphenoidal cephalocoeles usually present with spontaneous CSF rhinorrhoea (intermittent or continuous) without any previous history of trauma. Other symptoms may be fever with altered sensorium (symptoms of meningitis), visual blurring and headache (symptoms of raised intracranial hypertension). Nasal secretions can be tested for β2-transferrin, which is highly sensitive and specific for human CSF. The normal intra cranial pressure (ICP) is between 5 cm and 15 cm H20, which can be affected by diurnal and positional variations. An ICP consistently above 20 cm water is considered elevated and requires further evaluation.^7^ Examination of the CSF via lumbar puncture can be performed to assess for signs of meningitis and fundoscopic examination can evaluate for signs of papilloedema or optic atrophy.

Paranasal sinus CT (bone window) helps to localise the exact sites of the bony defects and assists in planning the approach for repair of the leak site. Imaging with CT cisternography is considered the gold standard examination for CSF leak evaluation as it determines both the bony defect and active contrast leak along with its lower cost, faster acquisition and easy availability when compared to MRI. After intrathecal administration of contrast material, the patient is positioned prone with the head in a dependent position prior to scanning. However, CT cisternography studies may be non-diagnostic if a patient has an intermittent CSF leak or does not have an active leak at the time of scanning.

On the contrary, MR cisternography does not require an active CSF leak to demonstrate the site. Detection of the CSF leak using MRI can be performed with either an unenhanced 3D-CISS sequence (three-dimensional constructive interference in steady state) or contrast-enhanced MR cisternography (CE-MRC) with a T1-weighted fat suppressed (T1W FS) sequence, which is an invasive technique. Imaging with 3D-CISS is a non-invasive and reliable technique and should be the first choice to localise the CSF leak as used in this study. It depends upon the presence of a defect at the bone-dura interface with demonstration of continuation of the hyperintense CSF signal into the PNS. It is dependent on the intrinsic T2 high signal of CSF for the detection of a CSF leak. Imaging with CE-MRC is helpful in conditions when there is no active leak or in complicated cases with a positive β2-transferrin measurement. On the T1W FS sequences, extravasation of contrast agent can be seen into the PNS or nasal cavity.^8^

Many spontaneous leaks may be associated with cephalocoeles and encephalocoeles, which can be confidently diagnosed on MRI; cephalocoeles are difficult to differentiate from mucosal thickening on CT. Details of the contents of the encephalocoele along with viability of the brain tissue can be assessed.^9^ Features of raised ICP such as arachnoid pits, prominent optic nerve sheath, empty or partially empty sella, prominent Meckel’s cave, and narrowing of the dural venous sinuses are identified with ease on MR imaging. The role of various imaging modalities in the detection of CSF leak has been listed in Table 1.

**TABLE 1:** The role of various imaging modalities.

Imaging modality	Diagnostic use
Unenhanced CT	Extent and pneumatisation of sphenoid sinus Arachnoid pits Osseous thinning Bony defects
CT cisternography	To demonstrate CSF leak To exclude a space occupying lesion causing raised ICP
MRI	Confirm encephalocoele Contents of encephalocoele Nature of herniated brain-healthy or gliotic Features of raised intracranial pressure Identify tiny encephalocoele missed on CT

CSF, Cerebrospinal fluid; ICP, intra cranial pressure.

Newer advances such as phase contrast MRI CSF flow studies can be used for the assessment of CSF flow abnormalities along with quantification of CSF flow parameters such as velocity, direction, and volume. It also plays a role in the assessment of efficacy and patency of ventriculoperitoneal (VP) shunts and ventriculostomy. This technique has so far been utilised in the evaluation of hydrocephalus (obstructive and non-obstructive), and posterior fossa malformations to ascertain communication of the posterior fossa cyst with adjacent CSF space.^10^ However, it has not yet been explored for the evaluation of CSF leaks through skull base defects.

### Management

The goals of management for intrasphenoidal encephalocoele are to halt ongoing CSF leakage, prevent recurrence of the CSF leak, and prevent intracranial complications (meningitis, tension pneumocephalus, cerebral abscess and subdural haematoma). Management is based on the aetiology of the leak, its location, frequency of the leak and flow volume.

Small, acquired defects in cases of closed head injury, resolve spontaneously and are managed conservatively unless there is neurological deterioration, or the defect is large. Large sphenoidal defects demand prompt surgical repair either via an endoscopic approach or by the traditional transcranial approach. Congenital defects are usually small defects with low volume and intermittent CSF leaks, which can be managed with bed rest, head elevation, and temporary CSF diversion to normalise the raised intracranial pressure.

For low volume and intermittent leaks, a conservative approach can be employed to reduce the raised intracranial pressure, by bed rest, head elevation, medications such as acetazolamide, IV mannitol and hypertonic saline. However, this approach has low success rates and high chances of recurrence and complications. For high volume or high frequency leaks, surgery has to be performed as soon as possible to avoid the risks of meningitis and intracranial hypotension. According to the Monroe Kelly doctrine, alterations in CSF volume because of a leak lead to disturbance in equilibrium between the brain parenchyma, intracranial blood and CSF volume with resultant compensatory dilatation of intracranial vascular spaces. Features of intracranial hypotension such as orthostatic headache (exacerbated on standing or straining) are more common with spinal CSF leaks than skull base leaks, explained by the concept of the hydrostatic indifference point.^11^ Imaging features of intracranial hypotension include diffuse pachymeningeal enhancement, subdural fluid collections, engorged venous sinuses, brain sagging, and few quantitative methods such as decreased mamillopontine distance and interpeduncular angle.^12^

Endoscopic access to the central part of the SS is easier and more convenient. Ethmoid and medial or perisellar types of sphenoid defects can be managed with the endonasal endoscopic approach. The nasal stage of the endoscopic approach is carried out by the otorhinolaryngology team while the sphenoid stage and final repair are performed by the neurosurgery team. The lateral extension of the SS (lateral recess) is usually difficult to access via the conventional endoscopic endonasal route. Hence, in these cases, the transcranial, extradural or intradural surgical approach is preferred. Intracranial infection complications are less frequent with the transcranial approach than the endoscopic approach (because of a potentially more sterile field). The pros and cons of the transcranial and endoscopic approaches have been enumerated in Table 2.

**TABLE 2:** Pros and cons of transcranial middle fossa repair and endoscopic repair.

Pros	Cons
**Transcranial middle fossa repair**	
Sterile operative field	Cosmetically inferior as scar may be evident
Easier to repair dural defect	More brain manipulation required
Dural defect and osseous defect can be addressed separately	Difficult to address medial and midline defects
Foreign glue can be avoided	
**Endoscopic approach**	
Scarless surgery	Unsterile corridor (nasal stage)
Extensive visualisation of the skull base and potential leak sites	It is difficult to individually address dural and osseous defects
Lesser brain manipulation	Foreign substances such as fibrin glue are required
	Difficult to address lateral recess defects

Patients refractory to medical management (i.e. have failed conservative management for 4 weeks or more), and having high volume leaks and complications are managed with surgery, which provides better long-term outcomes. Large bony defects and patients with normal CSF flow dynamics have more successful rates of CSF leak closure. Post-operative care is crucial in maintaining the graft integrity and good long-term prognosis. Common post-operative instructions with their rationale have been mentioned in Table 3.

**TABLE 3:** Post-operative management.

Post-operative instructions
Flat supine position for 72 h
Stool softeners
Cough suppressant and antihistaminic agent
Acetazolamide 250 mg TDS for 72 h
Lumbar drain
**Rationale**
To keep the CSF pressure minimum over the repaired site
To prevent raised ICP because of constipation
To prevent raised ICP because of cough and sneezing
To decrease CSF production
Only if there are any signs of CSF leak in the post-operative period

CSF, Cerebrospinal fluid; TDS, three times a day; ICP, intra cranial pressure.

## Conclusion

The emergence of lateral sphenoidal encephalocoele and associated CSF leaks is intricately linked to extensive lateral pneumatisation of the SS coupled with concomitant raised intracranial pressure, leading to osseous abnormalities. Imaging techniques such as CT cisternography and MRI are critical in characterising herniation, types of CSF leaks, understanding their underlying causes, and guiding effective management strategies.
